# Abuse and humiliation in the delivery room: Prevalence and associated factors of obstetric violence in Ghana

**DOI:** 10.3389/fpubh.2023.988961

**Published:** 2023-02-13

**Authors:** Abena Asefuaba Yalley, Dare Abioye, Seth Christopher Yaw Appiah, Anke Hoeffler

**Affiliations:** ^1^Department of Politics, Zukunftskolleg, University of Konstanz, Konstanz, Germany; ^2^Department of Politics and Public Administration, University of Konstanz, Konstanz, Germany; ^3^Department of Sociology and Social Work, Kwame Nkrumah University of Science and Technology, Kumasi, Ghana

**Keywords:** obstetric violence, facility-based childbirth, abuse, women, Ghana

## Abstract

**Background:**

Abuse and mistreatment of women during childbirth is a major barrier to facility-based delivery, putting women at risk of avoidable complications, trauma and negative health outcomes including death. We study the prevalence of obstetric violence (OV) and its associated factors in the Ashanti and Western Regions of Ghana.

**Methodology:**

A facility-based cross-sectional survey was conducted in eight public health facilities from September to December 2021. Specifically, close-ended questionnaires were administered to 1,854 women, aged 15–45 who gave birth in the health facilities. The data collected include the sociodemographic attributes of women, their obstetric history and experiences of OV based on the seven typologies according to the categorization by Bowser and Hills.

**Findings:**

We find that about two in every three women (65.3%) experience OV. The most common form of OV is non-confidential care (35.8%), followed by abandoned care (33.4%), non-dignified care (28.5%) and physical abuse (27.4%). Furthermore, 7.7% of women were detained in health facilities for their inability to pay their bills, 7.5% received non-consented care while 11.0% reported discriminated care. A test for associated factors of OV yielded few results. Single women (OR 1.6, 95% CI 1.2–2.2) and women who reported birth complications (OR 3.2, 95% CI 2.4–4.3) were more likely to experience OV compared with married women and women who had no birth complications. In addition, teenage mothers (OR 2.6, 95% CI 1.5–4.5) were more likely to experience physical abuse compared to older mothers. Rural vs. urban location, employment status, gender of birth attendant, type of delivery, time of delivery, the ethnicity of the mothers and their social class were all not statistically significant.

**Conclusion:**

The prevalence of OV in the Ashanti and Western Regions was high and only few variables were strongly associated with OV, suggesting that all women are at risk of abuse. Interventions should aim at promoting alternative birth strategies devoid of violence and changing the organizational culture of violence embedded in the obstetric care in Ghana.

## 1. Introduction

Maternal mortality and morbidity remain a major global health challenge and a threat to women's lives worldwide. According to the World Health Organization (WHO) (1), 211 deaths occur in every 100,000 live births as a result of preventable causes associated with pregnancy and childbirth globally with 94% of these deaths occurring in developing countries. Sub-Saharan Africa alone accounts for two-thirds of all maternal deaths worldwide due to poor obstetric care and unskilled birthing/low institutional deliveries (1–4). Ghana ranks high with a maternal mortality ratio of 310 deaths per 100,000 births, which is far above the global target of 70 deaths per 100,000 births (5). The majority of these deaths are preventable through the provision of high-quality maternal and obstetric services. Increasing the number of skilled birth attendants (SBAs) has been a cornerstone of international efforts to reduce maternal mortality as demonstrable evidence reveals low skilled birth attendance to be closely associated with high maternal mortality (1, 6). Studies demonstrate that about 70% of maternal and neonatal deaths could be prevented if all deliveries are attended by SBAs (2).

Consequently, there have been intense efforts in Ghana to increase institutional deliveries by strengthening community-based health planning and services, and free healthcare services for pregnant women through the National Health Insurance Scheme since 2008. However, a good number of pregnant women still deliver without skilled health care service. The rate of skilled birth deliveries is between 54 and 63% compared to the 80–97% of women who utilize antenatal care services (7, 8), indicating a high proportion of Ghanaian women who do not use facility-based services for childbirth, a likely contributor to the high maternal mortality ratio in Ghana. To reduce maternal mortality, there is a need to identify and address barriers that limit access and reduce the quality of obstetric services in the health system. Recent studies on the barriers of institutional birthing have established that experiences of mistreatment and abuse in health facilities are major impediments to their use (9–11). Across the globe, many women are abused during childbirth in health facilities. Although the global prevalence of OV is unknown, several studies have highlighted a gross of abuse and mistreatment associated with facility-based childbirth (12–16). The United Nations acknowledges that OV is widespread and systematic in nature (17).

Vacaflor (18) defines OV as “the violence exercised by health personnel on the body and reproductive processes of women (during pregnancy or childbirth), as expressed through dehumanizing treatment, medicalization abuse, and the conversion of natural processes of reproduction into pathological ones”. Obstetric violence is a relatively new concept in global health scholarship with scholars adopting different terminologies such as “mistreatment and abuse”, “disrespect and abuse” and “dehumanized care” to describe violence during childbirth. “Mistreatment and abuse” and “disrespect and abuse” helps to categorize distinctly the manifestations of violence while OV as a concept stresses the structural dimensions as a gender-based violence that intersects with institutional violence (19). All the terms emphasize the harmful impact of violence, the over medicalization of childbirth, violations of women's human rights and its gendered nature. For the purpose of this study, OV is used interchangeably with mistreatment and abuse.

WHO classifies mistreatment and abuse during childbirth to include:

“physical violence, humiliation and verbal abuse, intimidation, forced medical procedures, neglect, lack of confidentiality, failure to seek consent, refusal to administer pain medications, avoidable complications, refusal of medical admission, and detention of women after delivery based on their inability to pay medical bills” (20).

Negative experiences of obstetric care diminish incentives for institutional delivery and undermine technological equipment and facilities created to ensure optimal healthcare. The United Nations Educational, Scientific and Cultural Organization (UNESCO), in its Universal Declaration of Bioethics and Human Rights, declared that health does not depend solely on scientific and technological research developments, but also on psychosocial and cultural factors (21), thus, stressing the importance of humanized birthing. The WHO regulations on intrapartum care stress respectful and humanized care for all women which includes “dignity, privacy, and confidentiality, ensures freedom from harm and mistreatment and enables informed choice and continuous support during labor and childbirth” (22). OV counteracts this regulation by violating the bodily integrity of women, right to good health, respect, freedom from discrimination, privacy and choice (20, 23). It reduces women's satisfaction and trust in health facilities, which subsequently affects their willingness to give birth in facility-based services which provide proper management of birth-related complications. Recent studies on the challenges of facility-based delivery for women in developing countries found that obstetric violence is a significant barrier to utilizing health facilities for childbirth (14). In Latin American, women cite obstetric violence as the main reason for their failure to reuse health facilities for subsequent pregnancies, which leads to a considerable increase in maternal mortality and morbidity (12). Mistreatment and abuse also heighten trauma, which can lead to complications and poor health outcomes including death (24).

In recent years, a number of studies have reported on women's experiences of OV in some parts of the world, with a prevalence rate of 33% in Mexico, 44% in Argentina, 15% in India and 17% in the United States (25–28). Whereas, the phenomenon of OV is gaining attention in many countries, in Ghana, only few studies have been conducted on OV and these are mainly qualitative (10, 29, 30), limiting our understanding of the magnitude of OV among Ghanaian women. Studies that estimate the prevalence of OV are imperative for understanding the scope of this kind of violence based on which effective interventions that promote humanized care and minimize maternal mortality can be designed. We conducted a comprehensive literature review on OV and found that only two quantitative studies have been conducted in Ghana (31, 32). While these studies were useful in providing insight on the prevalence of mistreatment and abuse of women during childbirth in health facilities, they were based on a small sample size restricted to urban centers. Considering the fact that the majority of maternal deaths in Ghana occur among rural women due to low utilization of skilled birthing (43%) compared to 74% among urban women (33–35), there is a critical need for a comprehensive study that inculcates the experiences of rural women. Furthermore, there has been no quantitative study that estimates the prevalence and associated factors of obstetric violence in the Ashanti and Western Regions, the first and third most populous regions in Ghana, that are witnessing a decline in skilled birthing (7, 36). These gaps in knowledge could potentially impede efforts aimed at reducing maternal mortality in Ghana. In the current study, we employ a larger sample size of 1,854 mothers to examine the prevalence of obstetric violence, the associated factors and the characteristics of the perpetrators in urban and rural communities in Ashanti and Western Regions of Ghana.

## 2. Materials and methods

### 2.1. Study setting and design

A facility-based cross-sectional study was conducted from September to December 2021 in public health facilities in the Western and Ashanti Regions of Ghana. According to the Population and Housing Census conducted in 2021, the Western and Ashanti Regions have ~8 million inhabitants, which corresponds to 25.9 % of the total population (36). About 75% of women in Ashanti Region and 85% of women in the Western Region use antenatal care services while 53.4% and 53.8% of births take place in health facilities in Ashanti and Western Regions, respectively (7). The healthcare system in Ghana is classified into three main levels- primary, secondary and tertiary (37). The primary and secondary level healthcare services are the main points of delivery for most women in Ghana while the tertiary hospitals handle pregnancy and delivery-related complications. Eight health facilities were purposefully selected in urban and rural areas. The health facilities were included if they were public health facilities, were primary or secondary-level health facilities, provided obstetric care and maternal services, and had a high client flow for maternity services. In Ashanti Region, the study was conducted in two health facilities located in the Kumasi Metropolis district- the Maternal and Child Hospital and the Tafo Government Hospital—and two hospitals serving the rural communities—Nkenkaasu Government Hospital and Ejura District Hospital. Empirical data in Western Region was collected in the Kwesimintsim Polyclinic and Essikado Government hospital, the two main hospitals providing maternal care in Sekondi-Takoradi Metropolis and the Agona Nkwanta Health Center and Dixcove Government Hospital located in the rural part of the Western Region.

### 2.2. Study population and sampling

The survey involved a convenience sample of women who had given birth in the selected hospitals between January 2020 and December 2021 and accessed immunization services for their babies. The women were eligible to participate if they had given birth in the selected hospitals, were 15 years or older, were residents in either Ashanti and Western region and gave their consent to participate. Women who delivered outside the selected health facilities, employees of the hospitals, those whose last birth had occurred more than 24 months before contact and women who declined participation or consent, were excluded from the study. The cross-sectional survey being reported precedes a violence reduction intervention (unreported) that is to be followed after this study and as such, the sample size estimates are influenced by the overall study design. The sample was estimated based on Cochran's statistical formula for cross sectional studies (38) with the assumption that 50% of women experience mistreatment and abuse during childbirth, 95% confidence level and a relative precision of 5%, 10% non-response rate. The minimum sample size for the study participants as guided by the sample size formula (see Supplementary material) was 1,881. After cleaning the data from missing and incomplete entries, the final sample size was 1,854. When disaggregated by facility, the sample size for Maternal and Child hospital was 252, Tafo Government Hospital 275, Nkenkaasu government hospital 147, and Ejura district hospital 173. In the Western Region, the sample size for Kwesimintsim polyclinic was 291, Essikado government hospital 323, while the final sample size for Dixcove government hospital and Agona Nkwanta health center were 168 and 205, respectively.

### 2.3. Data collection procedure

We specially hired and trained enumerators for the recruitment of participants and the data collection. Recruiting was done in person at the child immunization centers where women were receiving immunization services for their babies. The women were approached by the enumerators and invited to participate in the study after a brief description of the study. The women who met the eligibility criteria were then provided with comprehensive information on the purpose of the study and those who agreed and gave their consent were enrolled. A structured, close-ended questionnaire was administered for the women digitally using the Survey-To-Go data collection tool that allowed us to conduct surveys offline or online. Questionnaires were administered in English or Akan, depending on the participant's preference. The recruitment of respondents continued until the sample size for each health facility was reached. The outcome variable was OV and was measured by the proportion of respondents who reported at least one form of abuse during their last pregnancy and childbirth. In the absence of a validated questionnaire for OV we designed our own questionnaire, closely based on the seven performance indicators developed by Bowser and Hill (39). These include physical abuse, non-consented care, non-confidential care, non-dignified care, discrimination, abandonment of care, and detention in facilities. The questionnaire was shared with public health experts for their critical review and a pilot test was conducted with the target population. In total, 35 verification criteria were utilized to measure the indicators of obstetric violence in a composite scale. The fieldwork was monitored throughout the data collection period, meaning that data entry checks were made every day to ensure consistency and reduce errors.

### 2.4. Ethical considerations

Ethical approval for this study was obtained from the Ethics Committee of the University of Konstanz, Germany (IRB Statement 37/2021) and the Ghana Health Service Ethics Review Committee (GHS-ERC 010/06/21). Further administrative consents were sought from all the directors of medical services in all the hospitals and health centers where the study was conducted. In addition to these, individual consents were granted by all the women who participated in the study before the administration of the questionnaire. The purpose of the study was duly explained to all women and individual consent forms were signed or thumb printed by respondents after a presentation of the information sheet (explaining the purpose of the study, confidentiality, duration of interviews, withdrawal of consent) were made. A translation of the information sheet into Akan was provided. Parental consent was sought for teenage mothers who participated in the study. Some respondents opted to give verbal consent. To ensure confidentiality, individual details such as the names and telephone numbers of all women were not collected.

### 2.5. Outcome variables

The survey instrument included questions on seven separate categories of OV: non-dignified care, non-consented care, discriminated care, non-confidential care, neglected care, detention in the health facility and physical abuse, which were all based on respondent's last childbirth experience. For each category, there were several verification criteria which had “Yes” or “No” dichotomized responses. An abuse was considered to have occurred for the specific category if a respondent reported “Yes” to any of the verification criteria under that category. The research instrument included questions on physical violence such as beating, pinching, holding of mouth/legs, stitching without anesthesia and slapping. Within the scope non-dignified care, respondents were asked if they had been verbally abused, shouted or yelled at, mocked, blamed, if their sexual life was disrespected or if they have received offensive criticism or remarks from health workers. Other aspects of the questionnaire asked questions about women's experiences of discriminatory treatments, if vaginal examinations or other medical procedures were conducted without their consent, if their privacy was breached by caregivers while performing vaginal examinations, or if delivery was carried out in the presence of others as well as if they had been ignored when they requested care or support. Finally, we also included questions on bribery, detention of women in health facilities for their inability to pay medical bills or bring the required materials. Prevalence rates were calculated for each category and for “any OV”.

### 2.6. Explanatory variables

The questionnaire also captured several demographic characteristics and obstetric history of respondents. The demographic variables included women's age, marital status, occupation, household income, level of education, the number of children, religion and education. Variables on women's obstetric history included antenatal attendance, the time of delivery, type of delivery (vaginal or cesarean section), facility of birth, the sex and qualification of the birth attendant and finally the presence of complications during labor or childbirth.

### 2.7. Data processing and analysis

The data were exported to IBM SPSS Statistics Version 28.0 for data processing. The analysis was carried out in two steps. First, we provide some descriptive analysis on the prevalence of obstetric violence before performing multivariate analyses between the potential associated factors and obstetric violence following the model by Bohren et al. (31). The Crude Odds Ratio (COR) and Adjusted Odds Ratios (AOR) were estimated with 95% confidence intervals (95% CI). All point estimates with a *p-value* < 0.05 were considered statistically significant.

## 3. Results

### 3.1. Sociodemographic characteristics and obstetric history of participants

Table 1 provides some descriptive data of the participants. The majority of women are aged 20–34 (75.6%), with teenagers making up a very small part of the sample (3.9%). The majority of women are married (72.3%) and 27.5% of all the mothers reported on the birth of their first child. A minority of the participants received no formal education (7.8%). More than half of the respondents (60.0%) live in a household with a monthly income of <500 Cedis (about 65 US Dollar). The majority of the respondents are Christians (80.0%) and they live in an urban setting (61.5%). Out of the eight health facilities, half were located in the Ashanti region. The majority of births were attended by a midwife (71.2%) and the presence of medical doctors was mostly reported for caesarian sections. The majority of the birth attendants were female (83.7%). Almost one in five women reported birth complications (18.9%) and caesarian sections accounted for one fifth of all deliveries (21.2%).

**Table 1:** Sociodemographic characteristics and obstetric history.

	**Rural (*****n*** = **713)**		**Urban (*****n*** = **1,141)**		**Total (*****n*** = **1,854)**		
	***N***	**%**	***N***	**%**	***N***	**%**	
**Maternal age (years)**							^***^
15–19 years	31	4.3%	41	3.6%	72	3.9%	
20–24 years	176	24.7%	171	15.0%	347	18.7%	
25–29 years	225	31.6%	358	31.4%	583	31.4%	
30–34 years	163	22.9%	310	27.2%	473	25.5%	
35–39 years	90	12.6%	204	17.9%	294	15.9%	
40–44 years	25	3.5%	51	4.5%	76	4.1%	
45 years and above	3	0.4%	6	0.5%	9	0.5%	
**Marital status**							
Single/never married	106	14.9%	191	16.7%	297	16.0%	
Married	510	71.5%	831	72.8%	1,341	72.3%	
Divorced	6	0.8%	5	0.4%	11	0.6%	
Widowed	2	0.3%	4	0.4%	6	0.3%	
Living with partner	89	12.5%	110	9.6%	199	10.7%	
**Education**							^***^
No formal education/schooling	94	13.2%	51	4.5%	145	7.8%	
Primary school (did not complete)	98	13.7%	49	4.3%	147	7.9%	
Primary school (completed)	94	13.2%	58	5.1%	152	8.2%	
Junior high school (did not complete)	77	10.8%	79	6.9%	156	8.4%	
Junior high school (completed)	144	20.2%	346	30.3%	490	26.4%	
Senior high school (did not complete)	40	5.6%	86	7.5%	126	6.8%	
Senior high school (completed)	108	15.1%	285	25.0%	393	21.2%	
Tertiary education	58	8.1%	187	16.4%	245	13.2%	
**Number of births**							^***^
One	152	21.3%	358	31.4%	510	27.5%	
Two	203	28.5%	306	26.8%	509	27.5%	
Three	164	23.0%	243	21.3%	407	22.0%	
Four	88	12.3%	148	13.0%	236	12.7%	
Five and above	106	14.9%	86	7.5%	192	10.4%	
**Employment status**							^**^
Working in the formal sector	70	9.8%	178	15.6%	248	13.4%	
Working in the informal sector	487	68.3%	744	65.2%	1,231	66.4%	
Keeping house (Homemaker/Housewife)	50	7.0%	71	6.2%	121	6.5%	
Looking for work/ Unemployed	63	8.8%	94	8.2%	157	8.5%	
Schooling/learning a trade	43	6.0%	54	4.7%	97	5.2%	
**Household income**							^***^
< 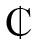 200 per month	182	25.5%	207	18.1%	389	21.0%	
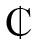 200– < 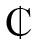 300	122	17.1%	173	15.2%	295	15.9%	
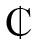 300– < 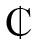 500	174	24.4%	254	22.3%	428	23.1%	
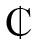 500– < 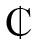 1,000	123	17.3%	299	26.2%	422	22.8%	
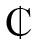 1,000– < 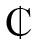 2,000	96	13.5%	155	13.6%	251	13.5%	
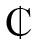 2,000– <5,000	14	2.0%	45	3.9%	59	3.2%	
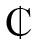 5,000 and above	2	0.3%	8	0.7%	10	0.5%	
Religion							^***^
Christian	496	69.6%	988	86.6%	1,484	80.0%	
Muslim	209	29.3%	150	13.1%	359	19.4%	
Traditional religion	8	1.1%	0	0.0%	8	0.4%	
Others	0	0.0%	3	0.3%	3	0.2%	
**Region**							
Ashanti	340	47.7%	527	46.2%	867	46.8%	
Western	373	52.3%	614	53.8%	987	53.2%	
**Name of facility**							^***^
Maternal and child hospital	0	0.0%	252	22.1%	252	13.6%	
Tafo government hospital	0	0.0%	275	24.1%	275	14.8%	
Nkenkaasu government hospital	167	23.4%	0	0.0%	167	9.0%	
Ejura district hospital	173	24.3%	0	0.0%	173	9.3%	
Kwesimintsim polyclinic	0	0.0%	291	25.5%	291	15.7%	
Essikado government hospital	0	0.0%	323	28.3%	323	17.4%	
Dixcove government hospital	168	23.6%	0	0.0%	168	9.1%	
Agona Nkwanta health center	205	28.8%	0	0.0%	205	11.1%	
**Asked for bribery**							^***^
Yes	67	9.4%	32	2.8%	99	5.3%	
No	646	90.6%	1,109	97.2%	1,755	94.7%	
**Type of delivery**							^***^
Caesarian section	91	12.8%	302	26.5%	1,461	78.8%	
Vaginal delivery	622	87.2%	839	73.5%	393	21.2%	
**Time of delivery**							
Day (6:00 am−6:59 pm)	425	59.6%	696	61.0%	1,121	60.5%	
Night (7:00 pm−5:59 am)	288	40.4%	445	39.0%	733	39.5%	
**Birth attendant**							^***^
Medical doctor (Gynecologist)	96	13.5%	303	26.6%	399	21.5%	
Midwife	528	74.1%	792	69.4%	1,320	71.2%	
Nurse	81	11.4%	45	3.9%	126	6.8%	
Community health nurse	8	1.1%	1	0.1%	9	0.5%	
**Sex of birth attendant**							
Male	102	14.3%	201	17.6%	303	16.3%	
Female	611	85.7%	940	82.4%	1,551	83.7%	
**Birth complication**							
Yes	145	20.3%	205	18.0%	350	18.9%	
No	568	79.7%	936	82.0%	1,504	81.1%	

Results with significant *p*-values (*p* < 0.05).

χ^2^ test used to compare demographic characteristics within settings ^*^p < 0.05,

** *p* < 0.01,

*** *p* < 0.001.

### 3.2. Prevalence and types of abuse during childbirth

In Table 2, we report the prevalence rates of OV. The majority of women reported that they experienced at least one form of OV (65.3%). The most common form of OV is non-confidential care or lack of privacy (35.8%), followed by neglected or abandoned care (33.4%), non-dignified care (28.5%) and physical abuse (27.4%). Detention in the health facility was relatively rare (7.7%) as was non-consented care (7.5%). There appears to be little difference by location as women in rural and urban areas report very similar rates.

**Table 2:** Prevalence of obstetric violence in rural and urban settings.

	**Rural (*****n*** = **713)**		**Urban (*****n*** = **1,141)**		**Total (*****n*** = **1,854)**	
	***N***	**%**	***N***	**%**	***N***	**%**
Any form of obstetric violence	453	63.50%	757	66.30%	1,210	65.30%
Non-confidential care/lack of privacy	234	32.80%	429	37.60%	663	35.80%
Neglected or abandoned care	208	29.20%	411	36.00%	619	33.40%
Non-dignified care	201	28.20%	327	28.70%	528	28.50%
Physical abuse	208	29.20%	300	26.30%	508	27.40%
Discriminated care	88	12.30%	116	10.20%	204	11.00%
Detention in the health facility	72	10.10%	70	6.10%	142	7.70%
Non-consented care	60	8.40%	79	6.90%	139	7.50%
**Non-confidential care/lack of privacy**	234	32.80%	429	37.60%	663	**35.8%** ^*^
Anyone other than the midwife or doctor present in the delivery room or labor room without consent	130	18.20%	254	22.30%	384	20.70%
Vaginal examinations were performed in the presence of other people	14	2.00%	86	7.50%	100	5.40%
Disclosure of medical information to others without your permission	18	2.50%	55	4.80%	73	3.90%
Not covered with any cloth or any screen to protect your privacy during delivery	115	16.20%	201	17.60%	316	17.00%
Disposing private information about you (loudly) to others	16	2.20%	77	6.70%	93	5.00%
**Neglected or abandoned care**	208	29.20%	411	36.00%	619	**33.4%** ^**^
Left unattended by midwives when you needed help	85	11.90%	146	12.80%	231	12.50%
Ignored when you requested for care	85	11.90%	152	13.30%	237	12.80%
Ignored when you ask questions	85	11.90%	123	10.80%	208	11.20%
Lack of support	72	10.10%	141	12.40%	213	11.50%
Health workers were unresponsive to your needs	80	11.20%	144	12.60%	224	12.10%
Separated your baby from you without medical justification	43	6.00%	94	8.20%	137	7.4%
Withdrawal of services for inability to provide materials	37	5.20%	33	2.90%	70	3.80%
First body contact with your baby was not performed	57	8.00%	148	13.00%	205	11.10%
Non dignified care	201	28.20%	327	28.70%	528	28.50%
Insults or verbal abuse	93	13.00%	124	10.90%	217	11.70%
Disrespect of my partner/spouse or family member	62	8.70%	60	5.30%	122	6.60%
Disrespect of my sexual life or history	15	2.10%	27	2.40%	42	2.30%
Laughed at me or made fun of me in a demeaning manner	16	2.20%	55	4.80%	71	3.80%
Criticized my personality, body, appearance	24	3.30%	91	8.50%	115	6.20%
Shouting/yelling	147	20.60%	189	16.60%	336	18.10%
Humiliation	51	7.20%	87	7.60%	138	7.40%
Scolding	16	2.20%	76	6.70%	92	5.00%
Blaming	28	3.90%	93	8.20%	121	6.50%
Offensive remarks	21	2.90%	133	11.70%	154	8.30%
Physical Abuse	208	29.20%	300	26.30%	508	27.40%
Beating/hitting	20	2.80%	38	4.30%	58	3.20%
Pinching	19	2.70%	11	1.00%	30	1.60%
Slapping face/thighs/back	58	8.10%	93	8.20%	151	8.10%
Holding your legs	47	6.60%	35	3.10%	82	4.40%
Holding/ covering your mouth	1	0.10%	7	0.60%	8	0.40%
Stitching without anesthesia	98	13.70%	127	11.10%	225	12.10%
Restriction of movement without medical justification	43	6.00%	84	7.40%	127	6.90%
Restriction from reactions to pain/ forcing me to keep quiet when in pain	93	13.00%	129	11.30%	222	12.00%
Discriminated care	88	12.30%	116	10.20%	204	11.00%
Discriminated treatment based on tribe, socio-economic status, HIV/AIDS	88	12.30%	116	10.20%	204	11.00%
**Detention in the health facility**	72	10.10%	70	6.10%	142	**7.7%** ^**^
Detained in hospital for inability to pay bills	54	7.60%	51	4.50%	105	5.70%
Detained for inability to provide required materials	39	5.50%	23	2.00%	62	3.30%
Asked to sweep/ mop/ or do anything for inability to pay bills	1	0.10%	7	0.60%	8	0.40%
Non consented care	60	8.40%	79	6.90%	139	7.50%
Midwife/medical doctor did not seek approval before beginning any medical procedure on you	29	4.10%	61	5.30%	90	4.90%
Internal examinations (vaginal examinations etc.) performed without approval	51	7.20%	58	5.10%	109	5.90%

Results with significant *p*-values (*p* < 0.05).

χ^2^ test used to demographic characteristics within settings.

* *p* < 0.05,

** *p* < 0.01,

^***^p < 0.001.

Inspecting the different categories of OV provides the following insights. In the non-confidential care category, the most common complaint was that other people were present in the labor room without consent, 20.7% of all women reporting lack of privacy. In the neglected or abandoned care category the reasons for OV were manifold, ranging from being left unattended to ignoring requests of care to healthcare workers being unresponsive. Non-dignified care was mainly due to the shouting and yelling by staff (18.1%) and being insulted or verbally abused (11.7%). In the physical violence category, the most common complaint was stitching without anesthesia (12.1%).

### 3.3. Factors associated with obstetric violence in Ghana

We now turn to the investigation of which characteristics are associated with the risk of experiencing any form of OV. Although a number of studies on the prevalence of OV exist (10, 11, 26, 28), there is yet no standardized model to investigate the correlates of OV. Rather than offer yet another modeling attempt, we chose to follow the recently published study by Bohren et al. (31) in order to benchmark our results. As in their study, we find it very difficult to identify variables that are robustly correlated with the experience of OV. Our results in Table 3 suggest that single women are at higher risk of abuse as they are more likely than married women to experience OV (OR 1.6). None of the other characteristics, age, education and first birth, are statistically significant (column 1). We then carried out a number of additional analyses. Since many of our variables are correlated, we take one variable at a time. We first investigate whether rates of OV differ significantly according to facility and we find that rates are significantly higher in the Dixcove Government Hospital, Maternal and Child Hospital and Kwesimintsim Policlinic. We also find that women who were asked for a bribe were more likely to experience OV (column 3, OR 2.4). Women were also significantly less likely to experience OV if the birth was attended by a midwife or medical doctor, as opposed to a nurse or a community health nurse (column 4, OR 0.4 for midwifes and OR 0.5 for doctors). Women who are poorer or those who reported their household income to be below 500 Cedis, were also less likely to experience OV (column 5). Women who reported complications during childbirth were much more likely to report OV, they were twice as likely to report OV compared to women who did not report complications (OR 3.2, column 6). Christians were also more likely to report OV (column 7, OR 1.4,). We also investigated rural vs. urban location, gender of birth attendant, type of delivery (vaginal vs. Caesarian section), time of childbirth (day/night), the ethnicity of the mothers and their social class. None of these variables were statistically significant and we report these result in the Supplementary material. Furthermore, irrespective of which variable was added, the ORs in our baseline model (Table 3, column 1) remained qualitatively similar. Following Bohren et al. (31), we also investigate the different forms of OV in Table 4. Although age is generally not significant, we find that teenage mothers are much more likely to experience physical abuse when compared to women aged 30 and over (OR 2.6). Marital status appears to be significantly associated with neglected care, non-confidential care and non-dignified care, single women being at greater risk of experiencing these forms of OV. There is also some suggestive evidence that women with no formal education are at higher risk of experiencing detention, non-consented care and discriminated care. First birth was not statistically significant in any of the models.

**Table 3:** Multivariable Logistic regression models of the factors associated with any form of obstetric violence.

	**Model 1**	**Model 2**	**Model 3**	**Model 4**	**Model 5**	**Model 6**	**Model 7**
**Age**							
15–19 years	1.39	1.71	1.35	1.39	1.48	1.282	1.45
	(0.73–2.62)	(0.89–3.27)	(0.712–2.56)	(0.73–2.63)	(0.78–2.80)	(0.67–2.45)	(0.77–2.75)
20–29 years	0.89	0.97	0.88	0.87	0.92	0.88	0.91
	(0.72–1.10)	(0.77–1.20)	(0.71–1.08)	(0.71–1.08)	(0.74–1.14)	(0.71–1.09)	(0.74–1.13)
≥ 30 years	1 (ref)	1 (ref)	1 (ref)	1 (ref)	1 (ref)	1 (ref)	1 (ref)
**Education**							
No education	1.24	1.37	1.21	1.15	1.27	1.29	1.37
	(0.86–1.79)	(0.93–2.02)	(0.84–1.76)	(0.8–1.68)	(0.88–1.84)	(0.89–1.87)	(0.94–2.00)
At least some education	1 (ref)	1 (ref)	1 (ref)	1 (ref)	1 (ref)	1 (ref)	1 (ref)
**Marital status**							
Single	1.6	1.55	1.6	1.606	1.7	1.66	1.56
	(1.17–2.17)^**^	(1.13–2.13)^**^	(1.17–2.18)^**^	(1.18–2.19)^**^	(1.24–2.33)^***^	(1.21–2.27)^**^	(1.15–2.13)^**^
Other than single	1 (ref)	1 (ref)	1 (ref)	1 (ref)	1 (ref)	1 (ref)	1 (ref)
**Number of births**							
First Birth	1.13	1.17	1.15	1.15	1.123	1.14	1.13
	(0.89–1.45)	(0.91–1.52)	(0.9–1.48)	(0.9–1.48)	(0.88–1.44)	(0.88–1.46)	(0.88–1.44)
≥2 births	1 (ref)	1 (ref)	1 (ref)	1 (ref)	1 (ref)	1 (ref)	1 (ref)
**Facility**							
Maternal and child hospital		2.57					
		(1.7–3.88)^***^					
Tafo government hospital		0.76					
		(0.53–1.11)					
Nkenkaasu government hospital		0.90					
		(0.59–1.37)					
Ejura district hospital		0.97					
		(0.64–1.48)					
Kwesimintsim polyclinic		2.49					
		(1.67–3.7)^***^					
Essikado government hospital		1.08					
		(0.75–1.56)					
Dixcove government hospital		3.77					
		(2.3–6.18)^***^					
Agona Nkwanta health center		1 (ref)					
**Asked for bribery**							
Yes NO			2.36				
			(1.42–3.94)^***^				
			1 (ref)				
**Birth attendant**							
Midwife				0.45			
				(0.29–0.69)^***^			
Medical doctor (Gynecologist)				0.48			
				(0.3–0.76)^**^			
Nurse/community health nurse				1 (ref)			
**Income level**							
<500 cedis					0.77		
					(0.63–0.95)^*^		
500 cedis and above					1 (ref)		
**Complications during birth**							
Yes						3.19	
						(2.36–4.3)^***^	
No						1 (ref)	
**Participant's religion**							
Christianity							1.38
							(1.08–1.76)^**^
Other religion							1 (ref)

Coefficients are odds ratios (95% CI). Results with significant *p*-values (*p* < 0.05) are indicated.

^y^*p* = 0.10,

* *p* < 0.05,

** *p* < 0.01,

*** *p* < 0.001.

This includes married, divorced, widowed, or living with partner.

**Table 4:** Multivariable Logistic regression models of the factors associated with different forms of obstetric violence.

	**Any form of OV**	**Physical abuse**	**Detention**	**Neglected**	**Non-confidential**	**Non-consented**	**Non-dignified**	**Discrimination**
**Age**								
15–19 years	1.39 (0.73–2.62)	2.58 (1.48–4.47) ^***^	1.73 (0.73–4.07)	0.66 (0.37–1.18)	0.73 (0.41–1.29)	1.59 (0.65–3.89)	1.33 (0.75–2.36)	1.55 (0.71–3.37)
20–29 years	0.89 (0.72–1.10)	1.2 (0.96–1.51)	1.24 (0.84–1.82)	0.85 (0.69–1.05)	0.9 (0.74–1.12)	1.11 (0.76–1.64)	1.06 (0.84–1.32)	1.02 (0.74–1.41)
≥ 30 years	1 (ref)	1 (ref)	1 (ref)	1 (ref)	1 (ref)	1 (ref)	1 (ref)	1 (ref)
**Education**								
No education	1.24 (0.86–1.79)	1.28 (0.88–1.85)	1.65 (0.95–2.88)^†^	0.85 (0.59–1.23)	0.96 (0.67–1.37)	1.91 (1.12–3.24)^*^	1.19 (0.83–1.72)	1.58 (0.98–2.54)^†^
Some education	1 (ref)	1 (ref)	1 (ref)	1 (ref)	1 (ref)	1 (ref)	1 (ref)	1 (ref)
**Marital status**								
Single	1.6 (1.17–2.17)^**^	1.08 (0.79–1.46)	1.09 (0.67–1.79)	1.29 (0.96–1.72)^†^	1.47 (1.10–1.95)^**^	1.16 (0.7–1.92)	1.37 (1.02–1.85)^*^	1.39 (0.91–2.12)
Other than single	1 (ref)	1 (ref)	1 (ref)	1 (ref)	1 (ref)	1 (ref)	1 (ref)	1 (ref)
**Number of births**								
First Birth	1.13 (0.89–1.45)	1.12 (0.87–1.46)	1.16 (0.76–1.78)	0.97 (0.75–1.24)	0.81 (0.63–1.04)^†^	0.96 (0.61–1.5)	0.81 (0.62–1.05)	0.72 (0.49–1.07)
≥2 births	1 (ref)	1 (ref)	1 (ref)	1 (ref)	1 (ref)	1 (ref)	1 (ref)	1 (ref)

Data are odds ratio (95% CI). Results with significant *p*–values (*p* < 0.05) are indicated.

† *p* = 0.10,

* *p* < 0.05,

** *p* < 0.01,

*** *p* < 0.001.

This includes married, divorced, widowed, or living with partner.

## 4. Discussion

In this study, we examine the prevalence of obstetric violence and its associated factors in rural and urban areas in Western and Ashanti Regions in Ghana. Like other studies (11, 32, 40–42), we have found the prevalence of OV to be high in health care facilities, the majority of women reported the experience of at least one form of OV (65.3%). However, it is difficult to identify characteristics that make women more vulnerable to OV. We provide some evidence that women who are married, older and have some formal education are less likely to be subjected to OV, but the evidence is not robust across all models we have investigated for different forms of OV. Some of the additional variables we investigated suggest that nurses and community health nurses tend to be more violent toward their patients. Only 7.3% of all births were attended by nurses and community health nurses and these clearly provide worse care for women during birth. The increased risk of violence by nurses and community health nurses could be because they are not trained to provide delivery services but are forced to take up delivery services due to the shortage of midwives especially in rural areas. Hence, they are more likely to use force and abuse due to a lack of skill during birth attendance. Qualitative evidence on the drivers of mistreatments and abuse reveal that health workers view obstetric violence as an essential means to ensure a positive birth outcome for the babies and therefore abuse women to force delivery (43–45). While concerns for the safety of the baby may provide some explanations for abuses during labor and childbirth, this procedure has negative health consequences for the mothers and this could be long term. Furthermore, our study also found that single women were about 60% more likely than married women to be abused during childbirth, revealing how gender constructions of marriage shape women's treatment. Marriage is considered a symbol of responsibility, honor and a prestigious identity for Ghanaian women (46), earning married women more respect in society with single mothers being perceived as irresponsible and somewhat sexually immoral. Our finding is consistent with Bohren et al. (31) study on mistreatment during childbirth in Ghana, Guinea, Myanmar and Nigeria, where abuse was much higher among single mothers than married women.

A high proportion of women reported that they were shouted at and that they were verbally abused (29.9%). This was more likely if the birth was attended by a midwife than by a medical doctor (see Supplementary material). With a midwife patient ratio of 2.7 per 1,000 patients in Ghana (47), midwives work under undue stress which significantly influences how women are treated. The shouting and verbal abuse is a strong indicator of the inability to cope with stress in the delivery room (31, 48–50). Also, women who experience birth complications are at higher risk of abuse. This could be explained by the long duration of contact that women with complications have with caregivers and longer stays in the health facilities. Long stays in health facilities have been associated with violence especially when violence is inculcated in daily routines of care (51). Similar evidence was found in Ethiopia where women who faced complications in the labor and birthing process were 1.6 times more likely to be abused (52).

Many women report physical abuse (27.4%) and the most common violation is stitching without anesthesia (12.4%). A similar form abuse has been reported in Mexico (26) and Nigeria (40) although at a relatively lower rate of 4 and 9%, respectively. Stitching of the vagina without anesthesia is considered torturous, a human right abuse and against the WHO regulations on intrapartum care (22, 53, 54). Within the category of physical violence, teenage mothers were at a higher risk, indicating inequalities in the treatment of women during childbirth. Our finding is supported by other studies where teenage mothers were humiliated for their engagement in pre-marital sex (10, 31). Much of the literature on African health focuses on the rural and urban differences (55–59), but we could find no differences between OV in rural and urban health facilities in Ghana, contrary to the case in India and Ethiopia where women in urban areas reported more abuse than women residing in rural areas (12, 60). This could probably be due to the fact that violence during birth is institutionalized and normalized as part of maternal care irrespective of where the facilities are located. This has been supported by qualitative studies on delivery room violence, suggesting the normalization of violence in delivery services in Ghanaian health institutions (61, 62).

We found some evidence that women were not given the right treatment although it was given to others in the same facility. This discriminated care was reported by 11.0% of the women. Other studies on OV found relatively higher discriminatory practices (21%) in Ethiopia and 20% in Nigeria (40, 52). We attempted to investigate the correlates of discrimination further but found no evidence that women were discriminated against due to their ethnic group or social class. Being a member of a religious minority appeared to work in the favor of women, the majority, in this region Christians, were at higher risk of experiencing OV.

The picture emerging from our study is that women suffer great harm in the delivery room and that this should be addressed urgently. Our study offers some pointers. As nurses are unlikely to provide care which is free of violence, they should receive additional training before attending to births. Birthing puts women in their most vulnerable physical and mental state and medical staff should receive more training to understand the negative consequences of violence on mothers, the importance of comprehensive care for women's optimal health and that of their newborns. Institutional factors such as high patient to caregiver ratio and lack of medical equipment play a significant role in inducing stress which tends to contribute to abusive treatments. Reducing the workload of caregivers, recruiting more caregivers and providing adequate medical supplies and equipment are important steps deal with the problem. More crucially, there is also the need for structural changes which include training on dignified care, gender norms and underlying socio-cultural factors the shape obstetric violence. Like all forms of gender-based violence, enforcing legal frameworks for maternal care and legal actions against OV are important steps to dealing with this menace. Considering the high prevalence of OV, there is the need for further studies to interrogate institutional and professional interventions to reduce and prevent abuse. From our study, it is evident that sociocultural factors such as gender constructions play a role in shaping women's experiences, hence further studies into the gendered dynamics of obstetric violence in Ghana is recommended. In the present study we only had a few HIV positive women (1.2%) and found no effect of HIV status on OV (results are available upon request). As the sub-sample was too small to draw meaningful conclusions, future studies should examine OV among HIV positive women through purposive sampling.

## 5. Conclusion

To summarize, we found a high prevalence rate of OV but there are few significant correlates in our regression analysis. Thus, we cannot point to a group of women that are at particularly high risk and conclude that all women who deliver in any of the eight public health facilities studied in Ghana are at a substantial risk of experiencing OV. This explains the reluctance of women to deliver in facilities and undermines the Ghanaian government's efforts to persuade women to have their babies in health facilities. We also established that there was no significant difference in the experiences of rural and urban women, thus emphasizing the endemic nature of obstetric violence in Ghanaian health institutions. On the other hand, our study also shows that great progress has been made. Health care is free of charge and only a small number of women are asked for a bribe, once a common practice (63).

## 6. Strengths and limitations

Key strengths of our study include the collection of a large sample size and the inclusion of health facilities in rural areas, extending the generalization of our results to the experiences of rural women. To reduce the risk of biases, enumerators were predominantly non-medical staff trained for the interviews. Although studies of this nature could be affected by recall bias, Simkin has demonstrated through her studies that memories from childbirth last up to 20 years and even more if women experience violence (64, 65). Hence our study is unlikely to suffer from recall bias. However, the study was conducted in the health facilities and this might lead to a risk of underestimation, as women may underreport their experiences out of courtesy or social desirability. Nonetheless, we found OV to be a very serious issue that compromises the health of women in Ghana.

## Data availability statement

The original contributions presented in the study are included in the article/Supplementary material, further inquiries can be directed to the corresponding author.

## Ethics statement

The studies involving human participants were reviewed and approved by the Ethics Committee of the University of Konstanz, Germany (IRB Statement 37/2021) and the Ghana Health Service Ethics Review Committee (GHS-ERC 010/06/21). Written informed consent to participate in this study was provided by the participants' legal guardian/next of kin.

## Author contributions

AY: conceptualization and design of the study, funding acquisition, methodology, data monitoring, and manuscript preparation. AH: design of study, funding acquisition, and manuscript preparation. DA: data cleaning and analysis. SA: methodology. All authors reviewed the manuscript and the content has been approved for publication.
