# Supplementary material for: Abuse and humiliation in the delivery room: Prevalence and associated factors of obstetric violence in Ghana
Source: Front Public Health. 2023 Feb 13;11:988961. doi: 10.3389/fpubh.2023.988961 (PMC9968731; doi:10.3389/fpubh.2023.988961)
Supplement: Supplementary file 1 [file Data_Sheet_1.PDF]

SUPPLEMENTARY MATERIALS

APPENDIX 1: SAMPLING FORMULAR

Using a relative precision of 5%, with a critical value of the normal standard values of 95% confidence level which corresponds to 1.96 (Za = Critical value at a confidence interval of 95% being 1.96), a design effect of 2.4 and a power of 80% (Zb = Critical value at the power of 80% is 0.842) while we accounted for 10% non-response rate. The minimum sample size for the study participants is as guided by the sample size formula below;

$$n = \frac{\left(Z_\alpha + Z_\beta\right)^2 p(1-p)}{e^2}$$

$$n = 2.5 * (1.96 + 0.84)^2 * 0.50 * (1 - 0.50) \frac{}{(0.05)^2}$$

$$n = \frac{2.5 * 7.84 * 0.50 * 0.50}{0.0025}$$

$$n = \frac{2.5 * 1.96}{0.0025}$$

n = 2.4 * 784

n = 1881,

After cleaning the data from missing and incomplete entries, the final sample size was 1854.

APPENDIX 2: Multivariable logistic regression models to assess factors potentially associated with obstetric violence

	Model 1	Model 2	Model 3	Model 4	Model 5	Model 6	Model 7	Model 8	Model 9	Model 10	Model 11	Model 12	Model 13
**Age**													
15-19 years	1,385 (0.732 - 2.619)	1,705 (0.889 - 3.273)	1,350 (0.712 - 2.558)	1,387 (0.732 - 2.631)	1,478 (0.780 - 2.802)	1,282 (0.671 - 2.450)	1,453 (0.767 - 2.753)	1,397 (0.738 - 2.642)	1,434 (0.756 - 2.720)	1,390 (0.735 - 2.631)	1,388 (0.734 - 2.625)	1,451 (0.766 - 2.749)	1,432 (0.752 - 2.730)
20-29 years	0,893 (0.724 - 1.102)	0,965 (0.774 - 1.203)	0,877 (0.710 - 1.083)	0,874 (0.707 - 1.080)	0,919 (0.744 - 1.136)	0,876 (0.708 -1.085)	0,914 (0.740 - 1.129)	0,896 (0.726 - 1.106)	0,912 (0.737 - 1.129)	0,895 (0.725 - 1.106)	0,892 (0.723 - 1.101)	0,924 (0.747 - 1.142)	0,902 (0.729 - 1.114)
≥ 30 years	1 (ref)	1 (ref)	1 (ref)	1 (ref)	1 (ref)	1 (ref)	1 (ref)	1 (ref)	1 (ref)	1 (ref)	1 (ref)	1 (ref)	1 (ref)
**Education**													
No education	1,237 (0.856 - 1.788)	1,37 (0.934 - 2.022)	1,214 (0.839 - 1.756)	1,154 (0.795 - 1.675)	1,269 (0.877 - 1.836)	1,287 (0.886 - 1.869)	1,373 (0.941 - 2.004)	1,233 (0.853 - 1.783)	1,282 (0.882 - 1.862)	1,240 (0.858 - 1.794)	1,241 (0.858 - 1.794)	1,346 (0.926 - 1.958)	1,243 (0.859 - 1.796)
At least some education	1 (ref)	1 (ref)	1 (ref)	1 (ref)	1 (ref)	1 (ref)	1 (ref)	1 (ref)	1 (ref)	1 (ref)	1 (ref)	1 (ref)	1 (ref)
**Marital_Status**													
Single	1,596 (1.172 - 2.172)**	1,548 (1.125 - 2.131)**	1,598 (1.172 - 2.178)**	1,606 (1.178 - 2.190)**	1,702 (1.244 - 2.327)**	1,661 (1.213 - 2.274)**	1,564 (1.148 - 2.131)*	1,604 (1.178 - 2.184)*	1,591 (1.169 - 2.167)*	1,596 (1.172 - 2.173)*	1,599 (1.174 - 2.177)*	1,532 (1.123 - 2.090)*	1,606 (1.178 - 2.188)**
Other than single ¶	1 (ref)	1 (ref)	1 (ref)	1 (ref)	1 (ref)	1 (ref)	1 (ref)	1 (ref)	1 (ref)	1 (ref)	1 (ref)	1 (ref)	1 (ref)
**First_Birth**													
First Birth	1,134 (0.885 - 1.452)	1,17 (0.905 - 1.518)	1,152 (0.899 - 1.477)	1,153 (0.899 - 1.480)	1,123 (0.877 - 1.439)	1,136 (0.884 - 1.461)	1,126 (0.879 - 1.442)	1,127 (0.880 - 1.443)	1,108 (0.862 - 1.423)	1,130 (0.881 - 1.449)	1,130 (0.883 - 1.447)	1,140 (0.889 - 1.460)	1,143 (0.891 - 1.465)
≥2 births	1 (ref)	1 (ref)	1 (ref)	1 (ref)	1 (ref)	1 (ref)	1 (ref)	1 (ref)	1 (ref)	1 (ref)	1 (ref)	1 (ref)	1 (ref)
**Facility**													
Maternal and Child Hospital		2,57 (1.701 - 3.878)***											
Tafo Government Hospital		0,76 (0.527 - 1.108)											
Nkenkaasu Government Hospital		0,90 (0.593 - 1.365)											
Ejura District Hospital		0,97 (0.640 - 1.479)											
Kwesimintsim Polyclinic		2,49 (1.672 - 3.698)***											
Essikado Government Hospital		1,08 (0.748 - 1.555)											
Dixcove Government Hospital		3,77 (2.298 - 6.181)***											
Agona Nkwanta Health Centre		1 (ref)											
**Asked for Bribery**													
Yes			2,360 (1.415 - 3.938)***										
No			1 (ref)										
**Birth attendant**													
Midwife				0,449 (0.291 - 0.693)***									
Medical doctor (Gynaecologist)				0,484 (0.302 - 0.775)**									
Nurse/community health nurse				1 (ref)									
**IncomeLevel**													
Less than 500 cedis					0,772 (0.630 - 0.946)*								
500 cedis and above					1 (ref)								
**Did you experience any complications during birth?**													
Yes						3,187 (2.361 - 4.301)***							
No						1 (ref)							
**Participant_Religion**													
Christianity							1,381 (1.084 - 1.759)**						
Other Religion							1 (ref)						
**Gender of Birth Attendant**													
Male								1,208 (0.926 - 1.575)					
Female								1 (ref)					
**Residence**													
Rural									0,886 (0.723 - 1.086)				
Urban									1 (ref)				
**Type of delivery**													
Caesarian section										1,030 (0.812 - 1.307)			
Vaginal delivery										1 (ref)			
**Time of delivery**													
Night											1,138 (0.934 - 1.386)		
Day											1 (ref)		
**Participant_Tribe**													
Akan												1,472 (0.710 - 3.050)	
Other Ghanaian tribes												1,054 (0.498 - 2.231)	
Non-Ghanaian												1 (ref)	
**WorkingClass**													
Working													1,090 (0847 - 1.401)
Not working													1 (ref)

Data are odds ratio (95% CI).
Results with significant p values (p<0·05) are indicated. †p=0·10. *p<0·05. **p<0·01. ***p<0·001.
¶ This includes married, divorced, widowed, or living with partner.

Table 3: Multivariable logistic regression models to assess factors potentially associated with obstetric violence

APPENDIX 3: Shouting/yelling * Birth attendant

		Birth attendant			
		Midwife	Medical doctor (Gynaecologist)	Nurse/community health nurse	Total
**Shouting/yelling**	Yes	246	62	28	336
		73,2%	18,5%	8,3%	100,0%
	No	1058	329	105	1492
		70,9%	22,1%	7,0%	100,0%
	N/A	16	8	2	26
		61,5%	30,8%	7,7%	100,0%
Total		1320	399	135	1854
		71,2%	21,5%	7,3%	100,0%
